# LINC00998‐encoded micropeptide SMIM30 promotes the G1/S transition of cell cycle by regulating cytosolic calcium level

**DOI:** 10.1002/1878-0261.13358

**Published:** 2022-12-29

**Authors:** Jin‐E Yang, Wang‐Jing Zhong, Jin‐Feng Li, Ying‐Ying Lin, Feng‐Ting Liu, Hao Tian, Ya‐Jing Chen, Xiao‐Yu Luo, Shi‐Mei Zhuang

**Affiliations:** ^1^ MOE Key Laboratory of Gene Function and Regulation, School of Life Sciences Sun Yat‐sen University Guangzhou China

**Keywords:** cell cycle, G1/S transition, micropeptide, SMIM30, sORF

## Abstract

The biological functions of short open reading frame (sORF)‐encoded micropeptides remain largely unknown. Here, we report that *LINC00998*, a previously annotated lncRNA, was upregulated in multiple cancer types and the sORF on *LINC00998* encoded a micropeptide named SMIM30. SMIM30 was localized in the membranes of the endoplasmic reticulum (ER) and mitochondria. Silencing SMIM30 inhibited the proliferation of hepatoma cells *in vitro* and suppressed the growth of tumor xenografts and N‐nitrosodiethylamine‐induced hepatoma. Overexpression of the 5′UTR‐sORF sequence of *LINC00998*, encoding wild‐type SMIM30, enhanced tumor cell growth, but this was abolished when a premature stop codon was introduced into the sORF via single‐base deletion. Gain‐ and loss‐of‐function studies revealed that SMIM30 peptide but not *LINC00998* reduced cytosolic calcium level, increased CDK4, cyclin E2, phosphorylated‐Rb and E2F1, and promoted the G1/S phase transition and cell proliferation. The effect of SMIM30 silencing was attenuated by a calcium chelator or the agonist of sarco/endoplasmic reticulum calcium ATPase (SERCA) pump. These findings suggest a novel function of micropeptide SMIM30 in promoting G1/S transition and cell proliferation by enhancing SERCA activity and reducing cytosolic calcium level.

## Graphical abstract

*LINC00998*, a previously annotated lncRNA, encodes an ER‐localized micropeptide named SMIM30. The authors found that the peptide, but not the lncRNA, reduced cytosolic calcium level by enhancing SERCA activity, then increased CDK4, cyclin E2 and E2F1 levels, and consequently promoted the G1/S phase transition and cell proliferation. These data unravel a new function of SMIM30 in regulating cytosolic calcium and G1/S transition.

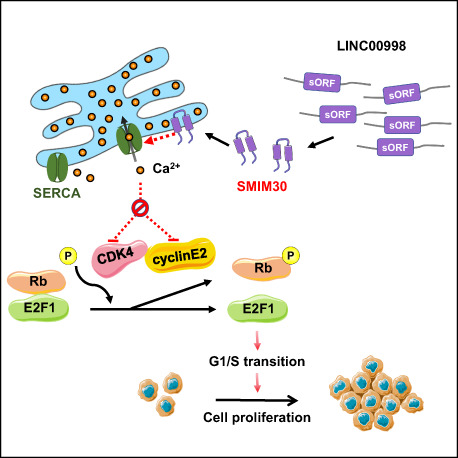

**Abbreviations**
DEN: N‐nitrosodiethylamine
ER: endoplasmic reticulum
GSEA: Gene Set Enrichment Analysis
HCC: hepatocellular carcinoma
qPCR: quantitative real‐time PCR
siRNA: small interfering RNA
SMIM30: Small Integral Membrane Protein 30
sORF: short open reading frame
TCGA: The Cancer Genome Atlas

## 1 Introduction

Recently, numerous short open reading frames (sORF) have been identified in transcripts that are previously defined as noncoding RNAs, such as primary microRNAs, circular RNAs, and long noncoding RNAs (lncRNAs) [1, 2]. Ribosome profiling revealed that a substantial portion of cytoplasmic lncRNAs that harbor evolutionary conserved sORFs were associated with ribosomes, suggesting their potential in encoding micropeptides [3, 4]. Owing to their small size and lack of known domains, only a handful of cellular micropeptides have been identified and assigned a function [5]. Emerging evidence has disclosed that micropeptides play essential roles in regulating diverse physiological processes [6], including stress response [7, 8], fatty acid metabolism [9], mitochondrial respiration [10, 11, 12], muscle differentiation, and performance [10, 13, 14, 15]. However, their functions in pathological processes, such as cancer development, remain largely unexplored [2].

Cell proliferation is a fundamental requirement for organism development and homeostasis. To ensure proper proliferation, the cell cycle, especially the G1 to S phase transition, is tightly regulated at many different levels. Defects in the cell cycle control, particularly dysregulation of G1/S transition, can ultimately lead to uncontrolled cell proliferation and tumorigenesis. The retinoblastoma (Rb) pathway, which consists of Rb, D‐ and E‐type cyclins, cyclin‐dependent kinases (CDKs) 4/6, CDK inhibitors and transcription factor E2F, plays critical roles in regulating the G1/S transition. Dysregulation of genes in the Rb pathway is common in various cancer types and has been shown to contribute to cancer development [16, 17, 18]. To date, only one micropeptide has been identified as the regulator of the G1/S transition [19].

Unlimited cell proliferation is one of the most important hallmarks of cancers. Understanding the development of cancers at the molecular level is urgently needed for identifying potential drug targets for cancer therapy. Given that many sORF‐containing lncRNAs are dysregulated in cancers, although their coding ability and functions have not yet been validated, it is expected that more micropeptides will emerge as important players in the etiology and progression of cancers.

In this study, we showed that a previously annotated lncRNA, LINC00998, encoded a conserved 59‐amino acid (aa) micropeptide. The peptide, predicted by UniProt Knowledgebase as an integral membrane protein, was named Small Integral Membrane Protein 30 (SMIM30) by HUGO Gene Nomenclature Committee. We further revealed that SMIM30 was located in the endoplasmic reticulum (ER) and mitochondria. Functional analysis showed that SMIM30, but not LINC00998, promoted the G1/S transition and proliferation of hepatoma cells by reducing the cytosolic calcium level. Moreover, SMIM30 was upregulated in multiple types of malignancies and silencing SMIM30 inhibited tumor growth *in vivo*. These findings highlight the importance of micropeptide SMIM30 in the G1/S transition and provide new insight into the regulatory network of the cell cycle control and tumor development.

## 2 Materials and methods

### 2.1 Tissue specimens and cell lines

Human HCC and adjacent non‐tumor tissues were collected from patients who underwent radical tumor resection at Sun Yat‐sen University Cancer Center, P.R. China. Both tumor and non‐tumor tissues were confirmed histologically. No local or systemic treatments were conducted before surgery, and no postoperative anti‐cancer therapies were administered prior to relapse. Informed consent was obtained from each patient, and the study was conformed to the standards set by the Declaration of Helsinki and approved by the Institutional Research Ethics Committee of Sun Yat‐sen University Cancer Center (Approval no. GZR2019‐086). Tissues were immediately snap‐frozen in liquid nitrogen until use.

The human embryonic kidney cell line HEK293T (CRL‐3216; ATCC, Manassas, VA, USA), human cervical carcinoma cell line Hela (CCL2; ATCC), and two human hepatoma cell lines HepG2 (HB‐8065, ATCC) and SK‐HEP‐1 (HTB‐52; ATCC) were cultured in Dulbecco's modified Eagle's medium (DMEM; Life Technologies, Gaithersburg, MD, USA) supplemented with 10% fetal bovine serum (FBS; Hyclone, Logan, UT, USA).

The stable cell lines were established by infecting SK‐HEP‐1 cells with lentiviruses that expressed the target sequence. These included sublines with stable expression of wildtype (WT) or frameshift mutant (FS) SMIM30 and their control line (Ctrl).

Two SMIM30‐knockout SK‐HEP‐1 sublines (SMIM30‐KO1 and KO2) were generated using CRISPR/Cas9 technology. In brief, a gRNA (5′‐TTGTGGAAGCAGTAGAAGC‐3′) targeting SMIM30 ORF was cloned into the PXPR_001 plasmid (#49535; Addgene, Watertown, MA, USA) and then transfected into SK‐HEP‐1 cells. Single‐cell colonies of puromycin‐resistant cells were expanded for 2 weeks, and SMIM30‐knockout sublines resulted from frameshift mutation were validated by genomic DNA sequencing and Western blotting. Parental SK‐HEP‐1 cell line with wildtype SMIM30 was used as a control.

### 2.2 RNA oligoribonucleotides and vectors

The small interfering RNAs (siRNAs) targeting different sites of the human SMIM30 (NM_001352688) were designed using the online tool siDESIGN (Dharmacon, Lafayette, CO, USA) and designated as siSMIM30‐1 and siSMIM30‐2. The negative control RNA duplex (NC) for siRNA is nonhomologous to any human genome sequence. All RNA oligonucleotides were purchased from RiboBio (Guangzhou, China).

**Fig. 1:**
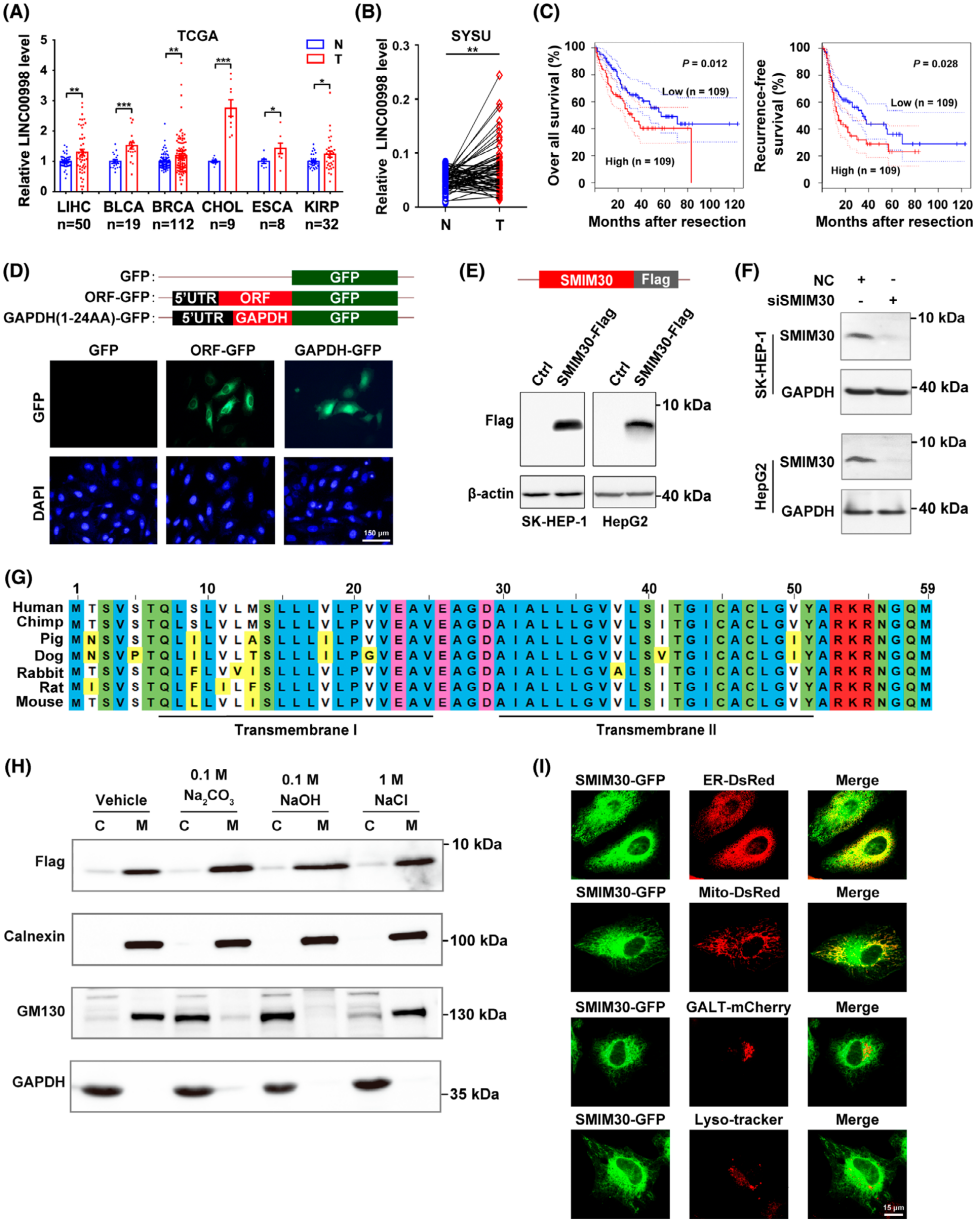
LINC00998‐Encoded transmembrane peptide is located in endoplasmic reticulum and mitochondria and is upregulated in multiple cancer types. (A) The levels of LINC00998 were elevated in multiple cancer types. Pan‐cancer analysis of LINC00998 expression in paired tumor (T) and non‐tumor (N) tissues was performed based on the cancer genome atlas (TCGA) data. LIHC, liver hepatocellular carcinoma (*n* = 50); BLCA, bladder urothelial carcinoma (*n* = 19); BRCA, breast invasive carcinoma (*n* = 112); CHOL, cholangio carcinoma (*n* = 9); ESCA, esophageal carcinoma (*n* = 8); KIRP, kidney renal papillary cell carcinoma (*n* = 32). Data are presented as mean ± SEM, *P < 0.05; **P < 0.01; ***P < 0.001 by two‐sided Student's *t*‐test. (B) The levels of LINC00998 were increased in HCC tissues. The levels of LINC00998 in 63 paired HCC (T) and adjacent non‐tumor (N) tissues from Sun Yat‐sen University cancer center (SYSU) were assessed by qPCR. **P < 0.01 by two‐sided Student's *t*‐test. (C) Upregulation of LINC00998 in HCC tissues was associated with worse overall survival and recurrence‐free survival. Analysis was performed using the transcriptome data of human HCC tissues derived from TCGA. Samples with LINC00998 levels in the top 30% (*n* = 109) and bottom 30% (*n* = 109) were defined as high‐ and low‐LINC00998 group, respectively. *P* values were determined by log‐rank test. The dotted lines represent the 95% confidence intervals (CI). (D) The predicted ORF on LINC00998 encoded a micropeptide. Hela cells were transfected with pc3.0‐ORF‐GFP(▵ATG), followed by staining with DAPI and photographed under a fluorescence microscope. pc3.0‐GFP(▵ATG) and pc3.0‐GAPDH‐GFP(▵ATG) were used as a negative and a positive control, respectively. Scale bar, 150 μm. (E) Expression of exogenous flag‐tagged SMIM30. SK‐HEP‐1 or HepG2 cells were transfected with pc3.0‐SMIM30‐flag for 48 h before Western blotting with anti‐flag antibody. β‐Actin, internal control. (F) Expression of cellular endogenous SMIM30 peptide. The indicated cells were transfected with negative control (NC) or siSMIM30 for 48 h before Western blotting with anti‐SMIM30 antibody. GAPDH, internal control. siSMIM30, a mixture of equal amount of siSMIM30‐1 and siSMIM30‐2 which target different regions of SMIM30 mRNA. (G) SMIM30 was highly conserved across mammalian species. Alignment of the predicted amino acid sequences for different mammalian SMIM30 was performed by constraint‐based multiple alignment tool (COBALT) from NCBI website. The amino acids in blue, green, pink, red, and bright yellow indicate the nonpolar, polar, negatively charged, positively charged, and non‐perfectly conserved amino acids, respectively. The two predicted transmembrane helical regions are underlined. (H) SMIM30 was a membrane‐integrated peptide. Homogenates of SK‐HEP‐1 cells that stably expressed flag‐tagged SMIM30 were untreated (vehicle) or incubated with the indicated reagents, and then centrifuged to yield the cytosol (C) or membrane (M) fractions, which were then subjected to immunoblotting analysis. Calnexin, an ER‐localized transmembrane protein, and GM130, a cis‐Golgi tethering protein, were used as the controls for membrane‐integrated molecule and membrane tethering molecule, respectively. (I) SMIM30 was localized in ER and mitochondria. Hela cells were cotransfected with the expression vector of SMIM30‐GFP and the expression construct of either ER‐DsRed, or Mito‐DsRed, or GALT‐mCherry, which marked ER, mitochondria, or Golgi body, respectively. The SMIM30‐GFP‐transfected cells were stained with lysoTracker (red) to mark lysosome. The cells were examined under a confocal microscopy. Scale bar, 15 μm. For D–F and H–I, data are representative of two independent experiments.

To verify the coding ability of LINC00998, the predicted coding sequence of SMIM30 (177 bp) together with its 5′‐UTR (150 bp) was fused in‐frame to the 5′‐end of the coding sequence of GFP (without ATG), inserted into the *Hind*III/*Bam*HI sites of pcDNA3.0 vector and named as pc3.0‐ORF‐GFP(▵ATG) (Fig. 1D). The pc3.0‐GFP(▵ATG) and pc3.0‐GAPDH‐GFP(▵ATG) were constructed as previously described and were used as negative and positive controls, respectively [20].

For transient expression of Flag‐ or GFP‐fused SMIM30, the pc3.0‐SMIM30‐Flag and pc3.0‐SMIM30‐GFP vectors were constructed. The coding sequence of human SMIM30 with a Flag tag or GFP coding sequence before the stop codon of SMIM30 was amplified by fusion PCR, then inserted into the *Bam*HI/*Eco*RI and *Hind*III/*Bam*HI sites of pcDNA3.0 vector, respectively.

For stable expression of Flag‐tagged wildtype (WT) or frameshift mutant (FS) SMIM30, pCDH‐SMIM30‐WT‐Flag, pCDH‐SMIM30‐FS‐Flag, and pCDH‐SMIM30‐Flag were constructed. To generate pCDH‐SMIM30‐WT, the coding sequence of SMIM30 together with its 5′‐UTR was fused with a Flag tag and cloned into the *Xba*I/*Bam*HI sites of pCDH‐CMV‐MCS‐EF1‐Puro vector (System Biosciences, Palo Alto, CA, USA). pCDH‐SMIM30‐FS was generated based on pCDH‐SMIM30‐WT, by deleting 1‐base pair in the second codon of SMIM30 which resulted in frameshift and premature stop codon. To construct pCDH‐SMIM30‐Flag, only the coding sequence of SMIM30 with a Flag tag was cloned into the *Xba*I/*Bam*HI sites of pCDH‐CMV‐MCS‐EF1‐Puro vector.

The fluorescence protein expression vectors, ER‐DsRed and Mito‐DsRed, were constructed as previously described [10] and used to label the ER and mitochondria in living cells, respectively. To obtain the GALT‐mCherry vector to label Golgi body, the coding sequence of 1–61 amino acids of human β1,4‐galactosyltransferase was fused in‐frame to the 5′‐end of the coding sequence of mCherry and cloned into the pcDNA3.0 vector.

All constructs were verified by direct DNA sequencing. All oligonucleotide sequences are listed in Table S1.

### 2.3 Cell transfection

Reverse transfection of RNA oligos was performed with Lipofectamine RNAiMAX (Invitrogen, Carlsbad, CA, USA). RNA duplex with a final concentration of 10–20 nm was transfected into SK‐HEP‐1 or HepG2 cell lines. Transfection of plasmid DNA was performed with Lipofectamine 3000 (Invitrogen).

### 2.4 Analysis of gene expression

The levels of RNA and protein were detected by quantitative real‐time PCR (qPCR) and Western blotting, respectively.

To detect the RNA levels of target genes, total RNA was extracted using TRIzol reagent (Life Technologies), then reverse‐transcribed with random primers (B0043; Sangon Biotech, Shanghai, China) using M‐MLV reverse transcriptase (M1701; Promega, Madison, WI, USA), followed by qPCR using 2× SYBR Green qPCR Master Mix (B21202; Bimake, Huston, TX, USA). All reactions were run in duplicate. The cycle threshold (*C*_t_) values differed by < 0.5 between the duplicates. The relative level of the target gene was normalized to that of the internal control gene, which yielded a $2^{-\Delta C_{t}}$ value. GAPDH or β‐actin was used as the internal control gene for the relative expression levels in tissues and cell lines.

The protein levels were detected by Western blotting. In brief, protein lysates were separated on an SDS‐polyacrylamide gel, transferred to polyvinylidene difluoride (PVDF) membranes (162–0177; Bio‐Rad, Hercules, CA, USA), and then sequentially incubated with primary and secondary antibodies. Immunoreactive signals were detected with an ECL kit (Thermo Fisher, Waltham, MA, USA). The antibodies used included: rabbit monoclonal antibodies against cyclin D1 (ab134175; Abcam, Cambridge, MA, USA), CDK4 (ab199728; Abcam), GM130 (ab52649; Abcam), Calnexin (ab133615; Abcam); rabbit polyclonal antibodies against phospho‐Ser780 of Rb (cat. 9307; CST, Boston, MA, USA), cyclin E2 (cat. 4132; CST), E2F1 (cat. 3742; CST); mouse monoclonal antibodies against Rb (cat. 9309; CST), CDK6 (cat. 3136; CST), β‐actin (BM0627; Boster, Wuhan, China), and Flag (F1804; Sigma‐Aldrich, St. Louis, MO, USA); mouse polyclonal antibody against GAPDH (BM1623; Boster).

### 2.5 Lentivirus production and infection

For lentivirus production, HEK293T cells were co‐transfected with a lentivirus expression vector and packaging plasmids (pMD2.G and psPAX2, Addgene). The lentiviral supernatant was harvested and stored in aliquots at −80 °C until use. Target cells were grown to 40% confluence and then infected with lentiviral supernatant supplemented with 10 mg·mL^−1^ Polybrene (Sigma‐Aldrich) for 72 h.

### 2.6 Rapid amplification of complementary DNA ends (RACE)

To amplify the 3′‐end of LINC00998, the total RNA from primary skin fibroblasts was subjected to reverse transcription with a 3′‐RACE‐adaptor primer, followed by nested PCR using gene‐specific primer and 3′‐RACE‐adaptor primer. The 5′‐end of the LINC00998 transcript was characterized using a SMARTer RACE 5′ Kit (634858; Takara, Kyoto, Japan) following the manufacturer's instructions.

### 2.7 Antibody generation

A custom polyclonal antibody against the C‐terminal region of SMIM30 was raised using the peptide sequence CLGVYARKRNGQM. Generation of the rabbit polyclonal anti‐SMIM30 IgG was performed by ABClonal (Wuhan, China).

### 2.8 Cell counting assay

Cell counting assays were used to evaluate cell proliferation *in vitro*. 1.5 × 10^4^ SK‐HEP‐1 or 2.5 × 10^4^ HepG2 cells were reversely transfected with NC or siSMIM30 and cultured in a 24‐well plate for the indicated periods before counting the cells by Count Star® Cell Analyzer (Alit Biotech, Shanghai, China).

### 2.9 Cell viability assay

SMIM30‐KO1, SMIM30‐KO2 and their parental SK‐HEP‐1 cells were seeded into a 96‐well plate at a density of 1500 cells per well. Cell viability was evaluated on the indicated time points using the Alarma‐Blue Kit (TL‐Yo56b; Telenbiotech, Guangzhou, China) according to the manufacturer's instructions. Briefly, cells were loaded with Alarma‐Blue solution, then incubated for 3 h at 37 °C before measuring the optical density at 590 nm using a microplate reader (Varioskan LUX Multimode Microplate Reader; Thermo Fisher).

### 2.10 Colony formation assay

Cells were reversely transfected with the indicated siRNA for 24 h, then reseeded in a 6‐well plate with a density of 250 (SK‐HEP‐1) or 300 (HepG2) cells per well and maintained in complete medium for 14 (HepG2) or 12 (SK‐HEP‐1) days. Colonies were fixed in methanol and stained with a 0.1% crystal violet solution for 15 min (min), washed three times with PBS, and then counted.

### 2.11 Cell cycle analysis

Cell cycle analyses were performed using detergent‐containing hypotonic solution Krishan's reagent [0.05 mg·mL^−1^ propidium iodide (PI), 0.1% sodium citrate, 10 mm NaCl, 0.02 mg·mL^−1^ RNase A (2158; TakaRa), 0.3% NP‐40] and fluorescence activated cell sorting (FACS; Gallios, Beckman Coulter, Miami, FL, USA), as reported [21]. Nuclear debris and overlapping nuclei were gated out.

### 2.12 EdU incorporation assay

DNA replication in the S phase was examined by 5‐ethylenyl‐2′‐deoxyuridine (EdU) incorporation assay (Cell‐Light™ EdU Apollo® 567 *In Vitro* Imaging Kit, Ruibo), and the number EdU‐positive cells relative to the total number of cells counted are presented. At least 500 cells were counted for each sample.

### 2.13 Mouse models

For mouse xenograft study, male NOD‐rkdcem26Cd52Il2rgem26Cd22/Nju (NCG) mice at 6–8 weeks of age were purchased from Nanjing Biomedical Research Institute of Nanjing University (Nanjing, China). SK‐HEP‐1 cells (4 × 10^6^) transfected with siSMIM30‐1 and siSMIM30‐2 mixture or negative control were resuspended in 100 μL of DMEM/matrigel (1:1 volume; 3432–005‐01 for matrigel; R&D Systems, Minneapolis, MN, USA) and subcutaneously injected into the right and left sides of the posterior flank, respectively. Mice were sacrificed 20 days after implantation. Tumor growth was measured every 3 days, and the volume of the tumor was measured with electronic digital calipers and calculated with the formula: volume = (length × width^2^)/2. At the end of the experiment, tumors were dissected, photographed, and weighed.

For the study of N‐nitrosodiethylamine (DEN)‐induced hepatocarcinogenesis, global SMIM30 knockout (SMIM30^−/−^) mice were generated by Cyagen Biosciences Inc. (Suzhou, China) on a C57BL/6N background using the CRISPR/Cas9 system. A founder with 127‐base pairs deletion in the coding region that created a frameshift mutation was chosen for this study [22]. SMIM30^−/−^ male mice (*n* = 5) at 3 weeks of age and their respective littermate control (WT, *n* = 3) were intraperitoneally injected once with 1 mg·kg^−1^ of DEN. Five weeks after the injection of DEN, CCl_4_ (0.2 mL·kg^−1^) was intraperitoneally administered twice a week for additional 14 weeks.

All mice were housed under a SPF condition (12‐h light/dark cycle, 50% relative humidity, between 25 and 27 °C) with free access to standard chow and tap water. All mouse experiments were approved by the Institutional Animal Care and Use Committee at Sun Yat‐sen University (SYSU‐IACUC‐2019‐B587 and SYSU‐IACUC‐2020‐000449). All procedures for animal experiments were performed in accordance with the Guide for the Care and Use of Laboratory Animals (NIH publications Nos. 80‐23, revised 1996) and according to institutional ethical guidelines for animal experiments of Sun Yat‐sen University.

### 2.14 Histology

Mouse livers from DEN‐treated mice were fixed in 4% formaldehyde, embedded in paraffin, and sectioned. Sections with 3.5 μm thickness were stained with hematoxylin and eosin (Zhong Shan‐Golden Bridge, Beijing, China) according to the manufacturer's protocol. Histology images were acquired with an Aperio Versa 200 Digital Scanner (Leica, Buffalo Grove, IL, USA) and examined for morphological alteration by two independent researchers.

### 2.15 Separation of cytosol and membrane components

Cytosol and membrane fractions were isolated from SK‐HEP‐1 cells stably expressing Flag‐tagged SMIM30. In brief, 2 × 10^7^ cells were washed twice with precooling PBS and resuspended in TNES buffer [50 mm Tris–HCl (pH 7.4), 150 mm NaCl, 5 mm EDTA, 250 mm sucrose, with protease inhibitors cocktail (B14012, Bimake)]. The cells were then homogenized using a 1‐ml syringe attached with a 0.45 mm needle for 45 passes. The unbroken cells, nuclei and mitochondria were removed by centrifugation at 3000 ***g***, 4 °C for 10 min. The cell homogenates were then untreated, or treated with either 0.1 m Na_2_CO_3_ or 0.1 m NaOH or 1 m NaCl for 15 min at room temperature before centrifugation at 120 000 ***g***, 4 °C for 1 h. The cytosol (supernatant) and membrane (pellets) fractions were, respectively, collected in lysis buffer containing protease inhibitor cocktail and then subjected to Western blotting.

### 2.16 Measurements of cytosolic calcium level

Acetoxymethyl ester fluorescent indicator (Fluo‐4/AM) was used to monitor the cytosolic calcium concentration in SK‐HEP‐1 and HepG2 cell lines. Briefly, cells at 90% confluence in a 12‐well plate were washed twice with PBS, then incubated with 4 μm Fluo‐4 AM dye diluted in serum‐free DMEM at 37 °C for 30 min, followed by washing once with PBS. Cells were incubated at room temperature for another 30 min to allow complete de‐esterification of intracellular AM esters. After washing three times with PBS, cells were harvested and resuspended in 300–400 μL PBS. The Fluo‐4 intensity, which represents the cytosolic calcium level, was detected by a microplate reader with an excitation wavelength of 488 nm, and the emission signal at 520 nm was collected.

### 2.17 Bioinformatics

The potential coding ability of lncRNAs and cross‐species conservation of sORF were evaluated using the online program ORFscore (http://www.sorfs.org) [23], which calculates an sORF score, a Floss score, and a PhyloCSF score for each lncRNA. Pan‐cancer analysis of LINC00998 expression in paired tumor and non‐tumor tissues was conducted using The Cancer Genome Atlas (TCGA) data, which was downloaded from the UCSC Xena (https://xena.ucsc.edu/public/). The transcriptome data across HCC samples in TCGA were subjected to Gene Set Enrichment Analysis (GSEA). Kaplan–Meier survival analysis for HCC patients with high or low gene expression level was performed through GEPIA website (http://gepia.cancer‐pku.cn/detail.php?).

### 2.18 Statistics

Data are expressed as the mean ± standard error of mean (SEM) from at least three independent experiments. Differences between groups were analyzed using an Student *t*‐test when only two groups were compared and one‐way ANOVA (analysis of variance) when more than two groups were compared. A *P* value of < 0.05 was considered statistically significant. All statistical tests were two‐sided and were performed using graphpad prism (GraphPad Software Inc., San Diego, CA, USA).

## 3 Results

### 3.1 LINC00998 encodes an ER‐ and mitochondria‐localized transmembrane micropeptide SMIM30

To discover the tumor‐associated micropeptides that were encoded by the annotated lncRNAs, we analyzed the ribosome profile (GSM1403307) of cycloheximide‐treated cells and identified nine ribosome‐bound intergenic lncRNAs that contained the predicted sORFs highly conserved across species (Fig. S1). Among them, only LINC00998 was abundantly expressed, while the others showed very low expression levels across different issues in TCGA data (Table S1). Subsequent analysis revealed that LINC00998 was significantly upregulated in various types of malignancies, including hepatocellular carcinoma (HCC) (Fig. 1A,B and Fig. S2A). Upregulation of LINC00998 in HCC tissues was associated with worse survival (Fig. 1C). Furthermore, LINC00998 had been predicted as a peptide‐coding RNA by the UniProt Knowledgebase and the corresponding peptide was named as SMIM30 by HUGO Gene Nomenclature Committee. Therefore, LINC00998 was selected for further study.

The subsequent characteristic analysis showed that the LINC00998 transcript contained 1004 nucleotides (nt) (Fig. S2B,C), and the corresponding gene was comprised of three exons that spanned ~ 1.83 kb (Fig. S2D). To test the translation capacity of the sORF on LINC00998 *in vivo*, we generated an expression construct in which the predicted ORF together with its 5′‐UTR was fused in‐frame to the 5′‐end of the coding sequence of GFP without start codon (SMIM30‐GFP). Substantial expression of the GFP‐fusion protein was detected in the cytoplasm of SMIM30‐GFP transfectants (Fig. 1D). Consistently, a specific band was detected for exogenous (Fig. 1E) and endogenous (Fig. 1F) SMIM30 at approximately 7 kDa by anti‐Flag antibody and by a customized polyclonal antibody against SMIM30, respectively. The data suggest that LINC00998 can encode a micropeptide SMIM30.

Bioinformatic analysis showed that SMIM30 contained 59 amino acids with two transmembrane helixes (residues 7–25‐aa and 30–52‐aa, Fig. S2E,F) and was highly conserved across mammalian species (Fig. 1G). Consistently, Flag‐tagged SMIM30 was detected in the membrane fraction no matter with or without alkaline treatment and showed the same pattern as calnexin, an ER transmembrane protein (Fig. 1H), but was different from the pattern of GM130, a cis‐Golgi tethering protein. These data suggest SMIM30 was as a membrane‐integrated peptide. To further verify the subcellular localization of SMIM30, the SMIM30‐GFP fusion protein was co‐expressed with different organelle markers. As shown, most of the SMIM30‐GFP was co‐localized with ER‐DsRed, a marker for ER, some was also co‐localized with mito‐DsRed, a mitochondrial marker (Fig. 1I). However, SMIM30‐GFP was not co‐localized with Golgi body or lysosome (Fig. 1I).

Collectively, these data indicate SMIM30 as a micropeptide located in the membrane of ER and mitochondria.

### 3.2 SMIM30 promotes cell proliferation by enhancing G1/S transition via the Rb pathway

**Fig. 2:**
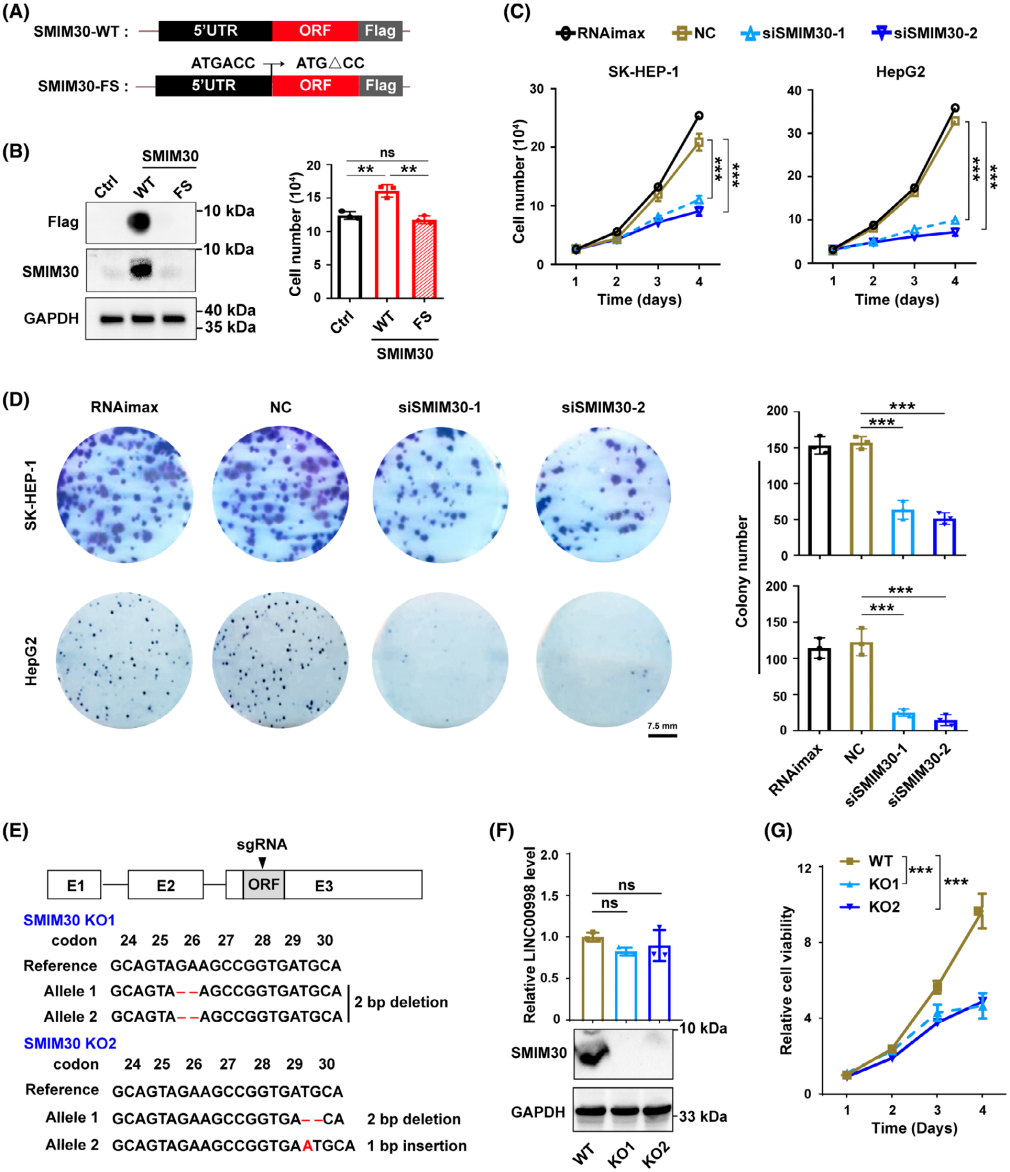
SMIM30 promotes proliferation of tumor cells *in vitro*. (A) Schematic showing the expression vectors of pCDH‐SMIM30‐WT‐flag (wildtype SMIM30) and pCDH‐SMIM30‐FS ‐flag (frameshift mutant SMIM30). (B) Ectopic expression of SMIM30 promoted hepatoma cell growth. SK‐HEP‐1 cells stably expressing wildtype (WT) or frameshift mutant (FS) SMIM30 or control (ctrl) were cultured in a 24‐well plate for 2 days before Western blotting (*left*), or cultured for 4 days before counting the cells (*right*). (C) SMIM30 knockdown inhibited the growth of hepatoma cells *in vitro*. SK‐HEP‐1 or HepG2 cells were transfected with NC or siSMIM30, then cultured for the indicated time periods before counting the cells. (D) SMIM30 knockdown inhibited the colony formation of hepatoma cells. SK‐HEP‐1 or HepG2 cells were transfected with the indicated siRNA for 48 h, then reseeded at low density in a 6‐well plate for 12 (SK‐HEP‐1) or 14 (HepG2) days before counting the colonies. RNAimax, cells exposed to Lipofectamine RNAiMAX without RNA duplex; NC, negative control of RNA duplex; siSMIM30‐1 and siSMIM30‐2, siRNAs targeting different regions of SMIM30 mRNA. Scale bar, 7.5 mm. (E) Construction of SMIM30 knockout SK‐HEP‐1 sublines. Top, scheme of the sgRNA and its targeting region (shown in black triangle). Bottom, sequence analysis of genomic DNA of the two SMIM30 KO cell lines (SMIM30 KO1 and KO2). Deletion or insertion of 1–2 base pairs resulted in frameshift mutation of SMIM30 ORF when translating. (F) SMIM30 peptide was knockout with retaining of LINC00998 RNA expression in the KO cell lines. The LINC00998 RNA and SMIM30 protein levels in the KO cells were detected by qPCR (upper) and Western blotting (lower), respectively. Parental SK‐HEP‐1 cells with wildtype SMIM30 were used a control (WT). (G) SMIM30 deficiency decreases cell viability. The SMIM30 WT and KO cell lines were seeded at low density in a 24‐well plate, then subjected to cell viability analysis using Alarma‐blue assay at the indicated time points. For B–D and F, G, data are represented as mean ± SEM of three independent repeats. *P* values were derived by one‐way ANOVA (B, D, and F) or two‐way ANOVA (C, G). ns, not significant; **P < 0.01; ***P < 0.001.

We then explored whether SMIM30 plays a role in cancer development. To verify that LINC00998 functioned through SMIM30 peptide but not lncRNA, we generated an expression construct containing full‐length LINC00998 with a Flag‐tag fused to its C terminus, and a frameshift mutant construct by introducing a single base deletion in the second codon of sORF to create a premature stop codon (Fig. 2A). Transfection of wildtype LINC00998 that expressed SMIM30 (Fig. 2B, *left*) increased cell number (Fig. 2B, *right*), but the frameshift mutant that could not expressed SMIM30 could not affect cell growth (Fig. 2B). Compared with control group, SMIM30‐silencing tumor cells (Fig. S3) displayed a significant reduction of cell growth (Fig. 2C) and colony formation (Fig. 2D). Consistently, two SMIM30‐knockout SK‐HEP‐1 sublines (SMIM30‐KO1 and KO2) with normal expression of LINC00998 RNA (Fig. 2E,F) showed reduced cell viability compared with SMIM30‐wildtype cell line (Fig. 2G).

**Fig. 3:**
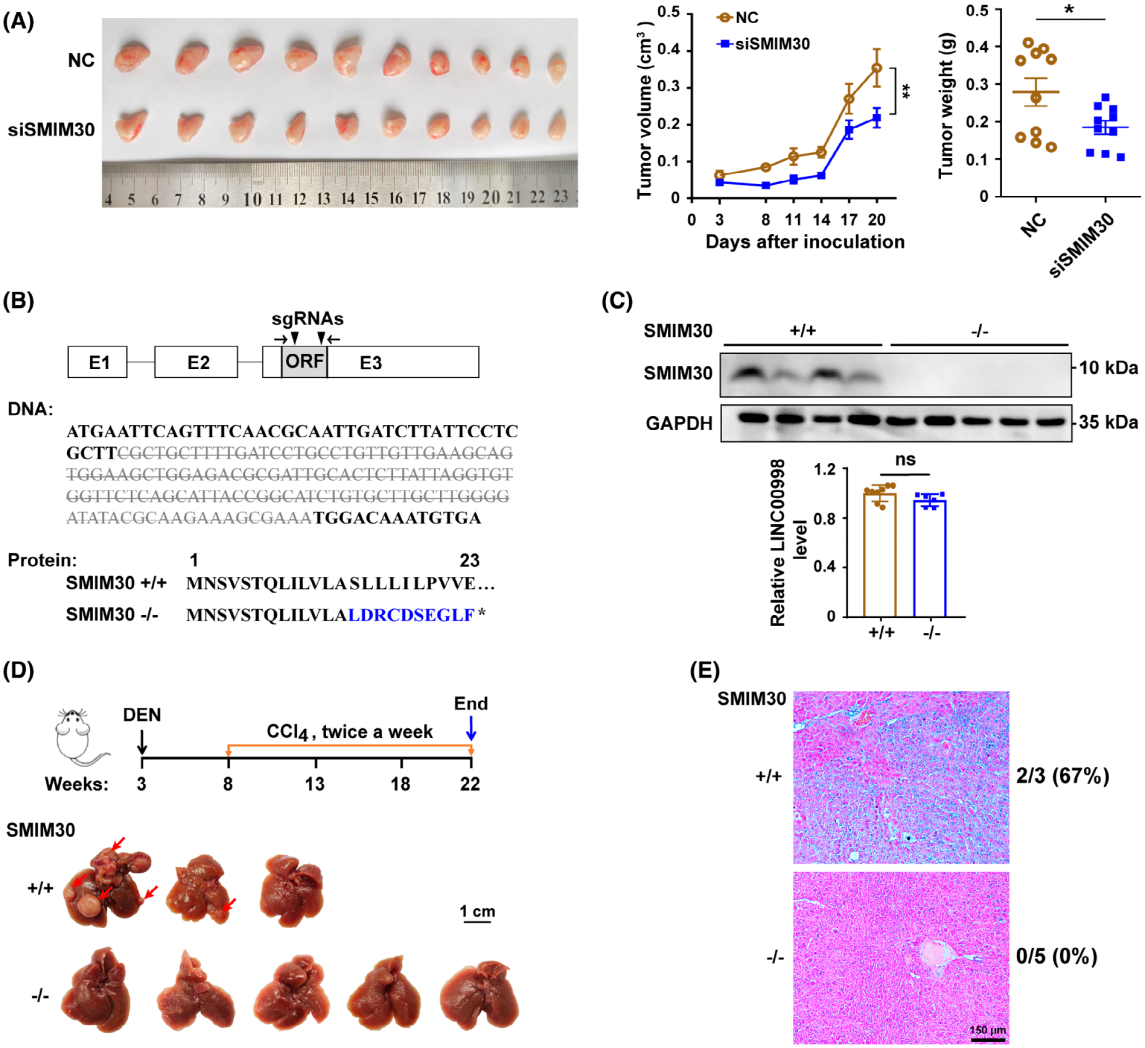
SMIM30 promotes tumor growth *in vivo*. (A) Silencing SMIM30 inhibited tumor growth *in vivo*. NC‐ or siSMIM30‐transfected SK‐HEP‐1 cells were subcutaneously implanted into NCG mice siSMIM30, a mixture of equal amount of siSMIM30‐1 and siSMIM30‐2. *n* = 10 mice per group. (B) Construction of SMIM30 KO (^−/−^) mice. Top, schematic diagram of the sgRNA‐targeting sites (shown in black triangles). Bottom, sequence analysis of SMIM30 genomic DNA for SMIM30^−/−^ mice revealed that a 127‐base pair deletion (gray strike through letters) in the coding region of SMIM30 resulted in a frameshift mutation. (C) SMIM30 peptide but not LINC00998 RNA was knockout in the SMIM30^−/−^ mice. The expression of SMIM30 peptide in the livers of wildtype SMIM30 (^+/+^) and SMIM30^−/−^ mice was detected by Western blotting (top, SMIM30^+/+^, *n* = 4; SMIM30^−/−^, *n* = 5), the LINC00998 RNA level was examined by qPCR using primers (shown in black arrows in B) flanking the sgRNA‐targeting sites (bottom, SMIM30^+/+^, *n* = 8; SMIM30^−/−^, *n* = 6). (D) Knockout of SMIM30 inhibited DEN‐induced hepatocarcinogenesis. Top, experimental design of the DEN‐induced HCC using SMIM30^+/+^ (*n* = 3) and SMIM30^−/−^ (*n* = 5) mice. Bottom, representative macroscopic view of livers. Arrows indicate the tumor nodules. Scale bar: 1 cm. (E) Representative H&E staining of SMIM30^+/+^ (*n* = 3) and SMIM30^−/−^ (*n* = 5) mouse livers. Tumor incidences are shown on the right of the image. Scale bar: 150 μm. For A and C, data are presented as mean ± SEM. *P* values were derived by two‐way ANOVA (A, tumor volume) or t‐test (A, tumor weight; C). ns, not significant; *P < 0.05; **P < 0.01.

We further evaluated whether SMIM30 affected tumor growth *in vivo*. Mouse xenograft models revealed that tumors derived from siSMIM30‐transfected SK‐HEP‐1 cells grew slower than control xenografts (Fig. 3A). In addition, we evaluated the role of SMIM30 in the development of DEN‐induced hepatoma using a SMIM30 knockout mice. As shown, 66.7% (2/3) of SMIM30‐wildtype mice developed tumors in the liver, whereas none (0/5) of SMIM30‐KO mice developed tumor (Fig. 3D,E). These results suggest that SMIM30 peptide, but not LINC00998 RNA, promotes cell proliferation and tumor growth.

**Fig. 4:**
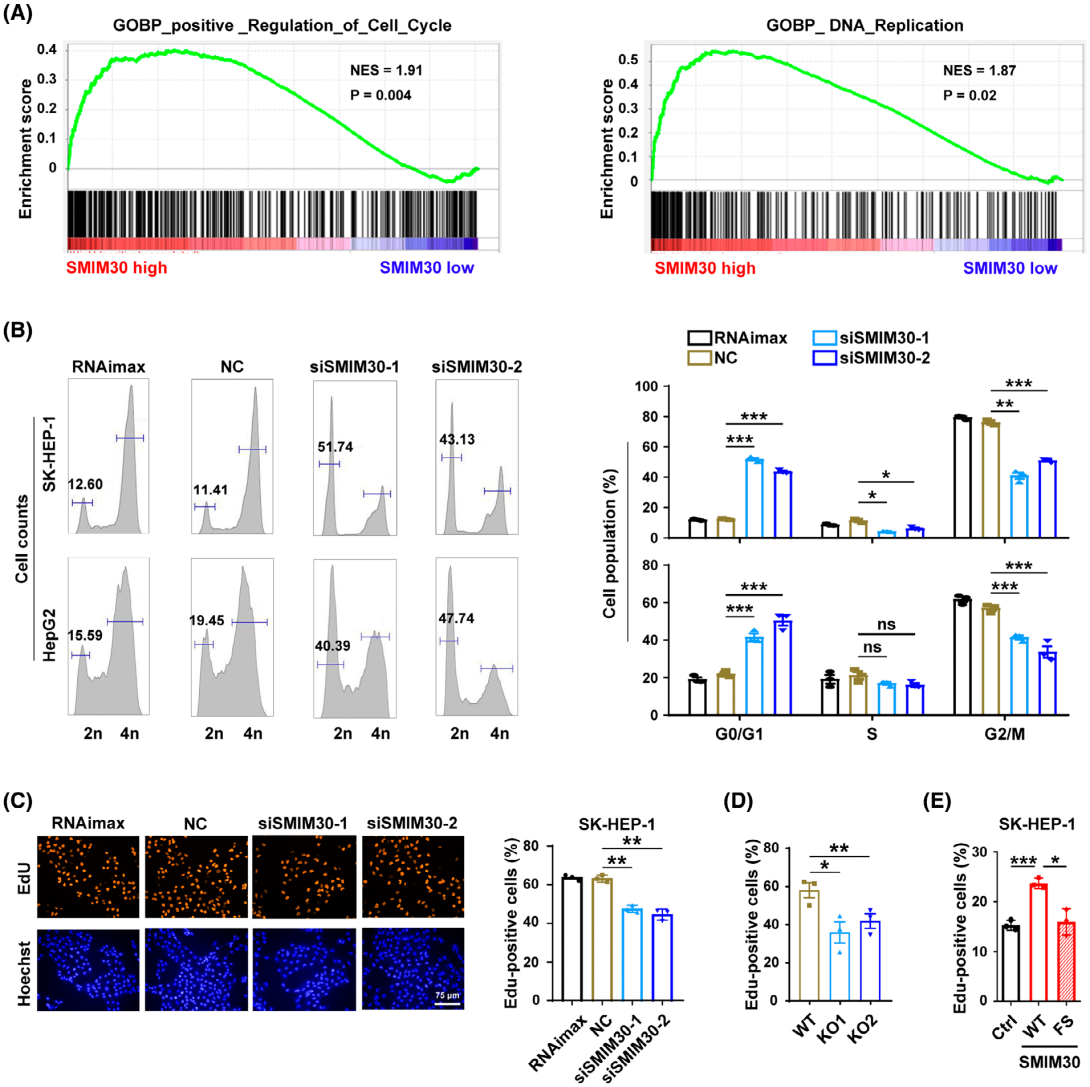
SMIM30 knockdown inhibits G1/S transition. (A) Genes positively regulating the cell cycle and genes in the DNA replication pathways were significantly enriched in HCC tissues with high SMIM30 level. Gene set enrichment analysis (GSEA) was performed using the transcriptome data of human HCC tissues derived from TCGA. The median mRNA level of the SMIM30 in all HCC tissues was chosen as the cut‐off value to separate the SMIM30 high‐level group (*n* = 186) from the low‐level group (*n* = 185). (B) Silencing SMIM30 induced a substantial increase in the G1 population. NC‐ or siSMIM30‐transfectants were treated with nocodazole for 12 h, which depolymerized the microtubules and blocked cell cycle at M‐phase, then subjected to FACS analysis of cell cycle. (C) Silencing SMIM30 inhibited DNA replication. NC or siSMIM30 transfected SK‐HEP‐1 cells were serum‐starved for 36 h, then cultured in 10% FBS‐containing medium for 15 h before EdU incorporation assay. RNAimax, cells exposed to Lipofectamine RNAiMAX without RNA duplex; NC, negative control of RNA duplex; siSMIM30‐1 and siSMIM30‐2, siRNAs targeting different regions of SMIM30 mRNA. Scale bar, 75 μm. (D) Knockout of SMIM30 inhibited DNA replication. The SMIM30 WT and KO cell lines were serum‐starved for 36 h, then cultured in 10% FBS‐containing medium for 15 h before EdU incorporation assay. (E) Ectopic expression of SMIM30 promoted DNA replication. SK‐HEP‐1 cells with stable expression of SMIM30‐WT (wildtype, WT), SMIM30‐FS (frameshift mutant, FS) or control (ctrl) were serum starved for 48 h, then cultured in 10% FBS‐containing medium for 12 h before EdU incorporation assay. At least 500 cells were counted for each sample. For (C–E), the number of EdU‐positive cells relative to the total number of cells counted are presented. For B–E, data are presented as mean ± SEM of three independent repeats. *P* values were derived by two‐way (B) or one‐way (C–E) ANOVA. ns, not significant; *P<0.05; **P < 0.01; ***P < 0.001.

To explore the molecular underpinnings of SMIM30 in tumor growth, we performed GSEA to identify the SMIM30‐associated pathways by using the transcriptome data of human HCC tissues derived from TCGA. The results revealed that genes positively regulating the cell cycle and DNA replication were significantly enriched in the tissues with higher SMIM30 level compared with those with lower SMIM30 level (Fig. 4A). We thus investigated whether SMIM30 affects cell cycle. Nocodazole‐synchronized cell models showed that knockdown of SMIM30 resulted in a substantial increase in the G1 population with a concomitant decrease of the S/G2/M population (Fig. 4B). EdU incorporation assays showed that the fraction of cells undergoing DNA replication was reduced by silencing (Fig. 4C and Fig. S4) or knockout (Fig. 4D) of SMIM30, while overexpressing wildtype but not frameshift mutant SMIM30 increased the number of DNA‐replicating cells (Fig. 4E). These data indicate that SMIM30 peptide promotes cell proliferation by enhancing the G1/S transition.

**Fig. 5:**
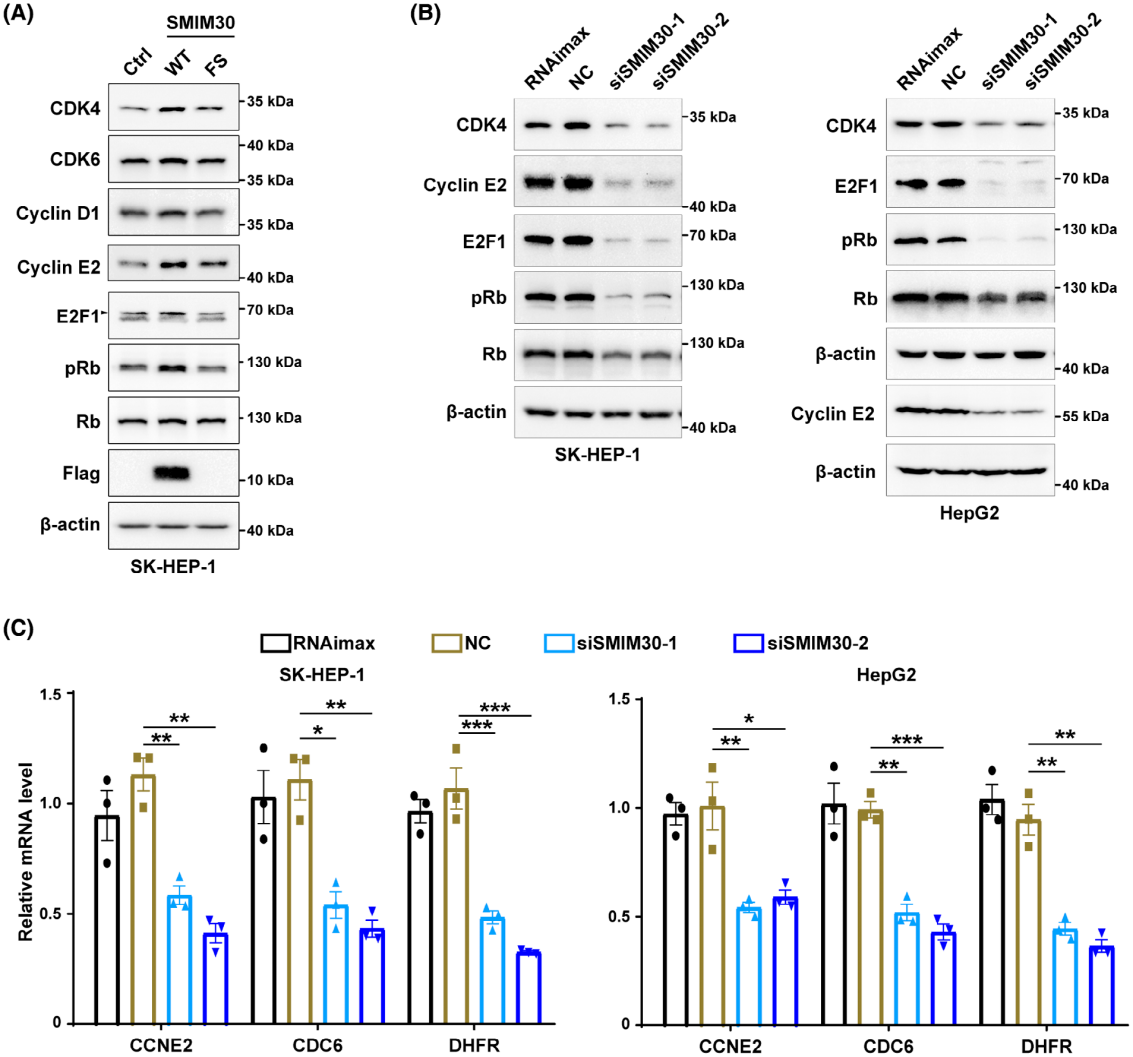
SMIM30 regulates the RB/E2F signaling. (A) Effect of SMIM30 overexpression on the protein levels of key regulators of G1/S transition. SK‐HEP‐1 cells stably expressing SMIM30‐WT (wildtype, WT), SMIM30‐FS (frameshift mutant, FS) or control (ctrl) were cultured for 48 h before Western blotting. (B) Silencing SMIM30 reduced the protein levels of CDK4, cyclin E2, E2F1 and phosphorylated RB (pRb). Black arrow indicates the E2F1 specific signal. Data are representative of two independent experiments. (C) Silencing SMIM30 reduced the mRNA levels of E2F target genes. SK‐HEP‐1 or HepG2 cells were transfected with NC or siSMIM30 for 36 h before Western blotting (B) or qPCR analysis (C). RNAimax, cells exposed to Lipofectamine RNAiMAX without RNA duplex; NC, negative control of RNA duplex; siSMIM30‐1 and siSMIM30‐2, siRNAs targeting different regions of SMIM30 mRNA. For C, data are represented as mean ± SEM from three independent repeats. *P* values were derived by one‐way ANOVA. *P < 0.05; **P < 0.01; ***P < 0.001.

We then explored whether SMIM30 affected the regulators of the G1/S transition. As known, Rb protein is phosphorylated by cyclin D/CDK4/CDK6 and then cyclin E/CDK2 complexes at the G1 phase, and the phosphorylated Rb (pRb) releases its bound E2F1, resulting in the transcription of E2F1 target genes and induction of the G1/S transition. We found that the protein levels of CDK4, cyclin E2, and E2F1, as well as pRb, were increased by overexpressing wildtype but not frameshift mutant SMIM30 (Fig. 5A), and their levels were reduced by silencing SMIM30 (Fig. 5B). In addition, the mRNA levels of E2F1 target genes, including CCNE2, CDC6, and DHFR, were reduced in SMIM30‐silencing cells (Fig. 5C). These results suggest that SMIM30 peptide, but not LINC00998 RNA, may promote the G1/S transition by regulating the cyclin/CDK‐Rb‐E2F1 pathway.

### 3.3 SMIM30 promotes G1/S transition by regulating cytosolic calcium level

**Fig. 6:**
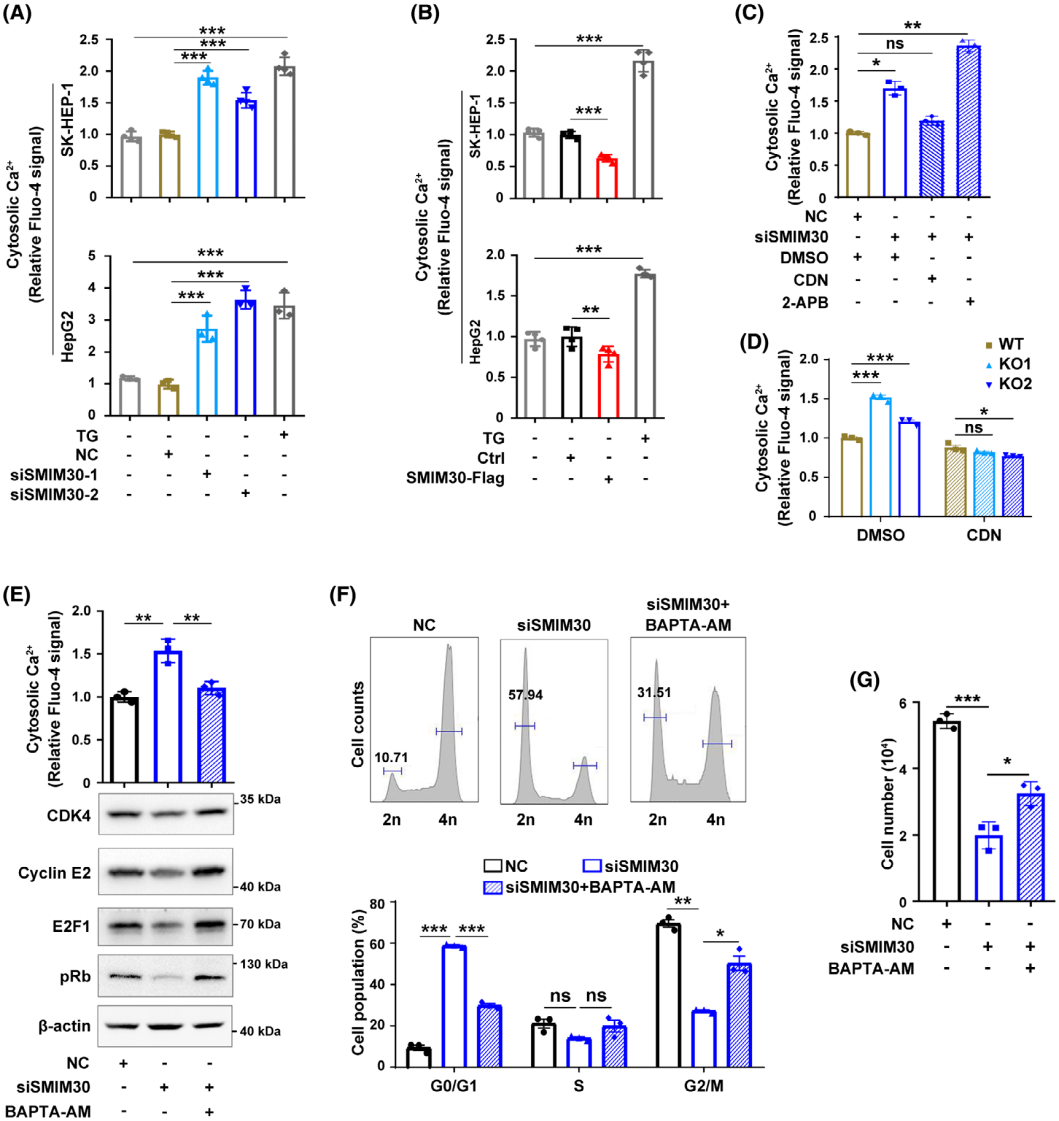
SMIM30 promotes the G1/S transition by regulating cytosolic calcium level. (A) Knockdown of SMIM30 increased the cytosolic calcium level. SK‐HEP‐1 or HepG2 cells were transfected with negative control (NC) or siSMIM30 for 48 h before detecting cytosolic calcium. siSMIM30‐1 and siSMIM30‐2, siRNAs targeting different regions of SMIM30 mRNA. (B) Overexpression of SMIM30 reduced the cytosolic calcium level. SK‐HEP‐1 or HepG2 cells stably expressing SMIM30‐Flag were cultured in a 24‐well plate for 48 h before detecting cytosolic calcium. Cells treated with 2 μm thapsigargin (TG, SERCA inhibitor) were used as a positive control. (C) SERCA agonist but not InsP3R inhibitor attenuated the effect of SMIM30 silencing in increasing cytosolic calcium level. siSMIM30 transfected SK‐HEP‐1 cells were treated with 10 μm CDN1163 (SERCA agonist) or 20 μm 2‐APB (InsP3R inhibitor) for 2 h before detecting cytosolic calcium. (D) ERCA agonist attenuated the effect of SMIM30 KO in increasing cytosolic calcium level. SMIM30 KO cells were treated with 10 μm CDN1163 for 2 h before detecting cytosolic calcium. Parental SK‐HEP‐1 cell line with WT SMIM30 was used as a control. (E) Treatment with calcium chelator abrogated the effect of siSMIM30 in reducing the levels of CDK4, cylinE2, E2F1 and pRb. NC‐ or siSMIM30‐transfectants were treated without or with 1 μm BAPTA‐AM (calcium chelator) for 36 h before cytosolic calcium detection or Western blotting (*n* = 2). (F) Exposure to calcium chelator abrogated the effect of siSMIM30 in repressing the G1/S transition. HepG2 cells were transfected with negative control (NC) or siSMIM30, followed by treatment without or with 1 μm BAPTA‐AM for 24 h before cell cycle analysis. (G) Treatment with calcium chelator ameliorated the role of siSMIM30 in inhibiting cell growth. NC‐ or siSMIM30‐transfectants were treated without or with 1 μm BAPTA‐AM for 3 days before counting cells. For (E–G), siSMIM30 represented a mixture of equal amount of siSMIM30‐1 and siSMIM30‐2. For A–G, data are represented as mean ± SEM of three independent repeats. *P* values were derived by one‐way (A–C, E, G) or two‐way (D, F) ANOVA. ns, not significant; *P < 0.05; **P < 0.01; ***P < 0.001.

Calcium serves as a second messenger for a number of critical cell activities, including proliferation. ER is the major calcium storage organelle that play crucial role in cytosolic calcium homeostasis [24, 25]. As SMIM30 was mainly located in ER, we thus examined whether SMIM30 exerted its function by regulating cytosolic calcium level. The results showed that silencing SMIM30 increased cytosolic calcium level (Fig. 6A), whereas overexpression of SMIM30 had the opposite effect (Fig. 6B). It is well known that calcium flux between ER and cytosol is mainly regulated through transmembrane receptors and calcium ion pumps on ER membrane [24]. Among them, inositol‐1,4,5‐triphosphate receptors (InsP3R) are responsible for calcium release from ER to cytosol, whereas the SERCA calcium pumps are responsible for transporting calcium from the cytosol to the ER. Notably, SERCA agonist but not InsP3R inhibitor attenuated the role of SMIM30 silencing or SMIM30 knockout in increasing cytosolic calcium level (Fig. 6C,D), suggesting that SMIM30 may function through SERCA. Moreover, treatment with BAPTA‐AM, a cytosolic calcium chelator, diminished the role of SMIM30 silencing in reducing CDK4, cyclin E2, E2F1 and pRb levels and in inhibiting the G1/S transition (Fig. 6E,F) and cell growth (Fig. 6G). These data suggest that SMIM30 peptide may promote the G1/S transition and cell growth by reducing cytosolic calcium level.

## 4 Discussion

Although exogenous designed peptites have been shown promising therapeutic potentials [26], our understanding on cellular endogenous micropeptides remains very limited. In this study, we found that LINC00998, a gene initially annotated as a lncRNA, encodes a membrane‐integrated micropeptide named SMIM30. SMIM30 was upregulated in various malignancies and it promoted the G1/S transition by reducing cytosolic calcium level, thereby enhancing cell proliferation and tumor growth.

Whether LINC00998 functions as a lncRNA or a micropeptide remains controversial. Studies in glioblastoma showed that LINC00998 RNA directly interacted with chromobox 3 protein (CBX3) and prevented it from ubiquitin‐mediated degradation, suggesting that LINC00998 functions as a noncoding RNA [27]. In contrast to general findings that most of the lncRNAs are poorly conserved [28], human LINC00998 contains a highly conserved sORF, and the deduced peptide SMIM30 shares more than 80% amino acid identity with its mammalian homologs. Here we showed that antibodies against the C‐terminus of SMIM30 recognized the cellular endogenous peptide. Besides, sequence and localization analyses disclosed that SMIM30 contained two hydrophobic transmembrane domains and was located in ER and mitochondria. Furthermore, overexpression of wildtype but not frameshift SMIM30 promotes cell proliferation, whereas knockout of SMIM30 peptide with retaining of LINC00998 RNA expression decreases cell viability, which together suggest that SMIM30 is a functional peptide, and LINC00998‐encoded SMIM30, but not LINC00998 RNA itself, exerts oncogenic roles in our study models. While this paper was under preparation, Pang and colleagues reported that LINC00998 RNA could bind to 40s ribosomal protein S6 (RPS6) and be translated into a micropeptide [29], which is consistent with our findings.

Maintaining intracellular calcium homeostasis is critical for cell survival. It is mainly regulated by extracellular calcium entry through calcium‐related channels on the plasma membrane, or by organelle calcium release [25]. It has been shown that increase in cytosolic and organelle calcium concentration can work as a signaling messenger for a wide array of cell activities [30, 31]. ER is the main calcium storage organelle that plays crucial role in regulating cytosolic calcium level. Calcium stored in ER can be released into the cytosol through transmembrane receptors, mainly InsP3R and ryanodine receptors (RyR). In contrast, SERCA, the calcium ion pump, is responsible for transporting calcium from cytosol to ER against the concentration gradient [25]. Dysregulation of ER calcium pump has been linked to various types of cancer [32, 33]. However, the exact roles of intracellular calcium on cell growth remain unclear. Seo *et al*. found that SERCA2 is upregulated in ovarian cancers. Moreover, curcumin, an anticancer agent, can cause a sustained increase of cytosolic calcium concentration by inhibiting SERCA activity, which results in cell apoptosis [34]. Consistently, small molecules targeting SERCA2 induced apoptosis of colon cancer cells [35]. Paradoxically, SERCA3 was significantly reduced in colon cancers, which may drive proliferation; thus, reintroducing SERCA3, in this case, would promote cell apoptosis [36]. In this study, we showed that SMIM30 is a novel ER‐located transmembrane peptide that reduced the cytosolic calcium level. Moreover, SERCA agonist attenuated the role of siSMIM30 in increasing cytosolic calcium level, suggesting that SMIM30 may function as an “on switch” for SERCA, which warrants further investigation.

To ensure proper proliferation and cell growth, the cell cycle is tightly regulated. Gain‐ and loss‐of‐function studies showed that SMIM30 reduced cytosolic calcium level, increased the protein levels of key components in the Rb pathway, including CDK4, cyclin E2, E2F1 and pRb, and promoted the G1/S transition and cell growth; and calcium chelator attenuated the effect of SMIM30 silencing, suggesting that SMIM30 may promote tumor cell growth by regulating intracellular calcium concentration. To be note, Pang et al. [29] also disclosed that SMIM30 promoted the proliferation of HCC cells, through interacting with non‐receptor tyrosine kinase SRC/YES1. Taken together, these findings suggest that SMIM30 may sustain cell proliferation through different signaling pathways.

## 5 Conclusions

In summary, we identified a micropeptide SMIM30 that was encoded by a previously annotated lncRNA LINC00998 and characterized SMIM30 as a novel regulator for both cytosolic calcium level and the G1/S transition, which provides new insight into the mechanisms of cell cycle control and tumor development.

## Conflict of interest

The authors declare no conflict of interest.

## Author contributions

JEY and WJZ designed the study and performed experiments, discussed and interpreted the data, and wrote the manuscript. JFL, YYL, HT, FTL, YJC, and XYL performed experiments and interpreted the data. SMZ supervised and designed the study, discussed and interpreted the data, and wrote the manuscript. All authors read and approved the final manuscript.

## Supporting information


**Fig. S1.** The screening workflow for candidate lncRNAs with peptide‐coding potential.
**Fig. S2.** Characterization of SMIM30 gene.
**Fig. S3.** Knockdown of cellular SMIM30 by siRNAs.
**Fig. S4.** Silencing SMIM30 reduced the fraction of cells with DNA replication.
**Table S1.** The levels of lncRNAs in different human tissues.
**Table S2.** Sequences of DNA and RNA oligonucleotides.

## Data Availability

The data that support survival analysis for HCC patients with high or low LINC00998 expression levels are openly available in TCGA and GEPIA website (http://gepia.cancer‐pku.cn/detail.php?gene=LINC00998). The expression data of SMIM30 in various tumors were obtained from the TCGA database and downloaded from the UCSC Xena database (https://xena.ucsc.edu/public/).
