# Supplementary material for: LINC00998‐encoded micropeptide SMIM30 promotes the G1/S transition of cell cycle by regulating cytosolic calcium level
Source: Mol Oncol. 2022 Dec 29;17(5):901–16. doi: 10.1002/1878-0261.13358 (PMC10158777; doi:10.1002/1878-0261.13358)
Supplement: Supplementary file 1 — Fig. S1. The screening workflow for candidate lncRNAs with peptide‐coding potential. Fig. S2. Characterization of SMIM30 gene. Fig. S3. Knockdown of cellular SMIM30 by siRNAs. Fig. S4. Silencing SMIM30 reduced the fraction of cells with DNA replication. Table S1. The levels of lncRNAs in different human tissues. Table S2. Sequences of DNA and RNA oligonucleotides. [file MOL2-17-901-s001.doc]

**Supplemental Information**

**LINC00998-encoded micropeptide SMIM30 promotes the G1/S transition of cell cycle** **by regulating cytosolic calcium level**

*Jin-E Yang**^*^, Wang-Jing Zhong, Jin-Feng Li, Ying-Ying Lin, Feng-Ting Liu, Hao Tian, Ya-Jing Chen, Xiao-Yu Luo, and Shi-Mei Zhuang^*^*

**Inventory of supplementary data**

1. Supplementary Figures and Figure Legends……….….……….………..…..Page 2 - 6

2. Supplementary Tables…………………………………………………………Page 7 - 9

3. Supplementary reference……………………….……………………......………Page 10

**Supplementary figures and figure legends**

**Figure S1**


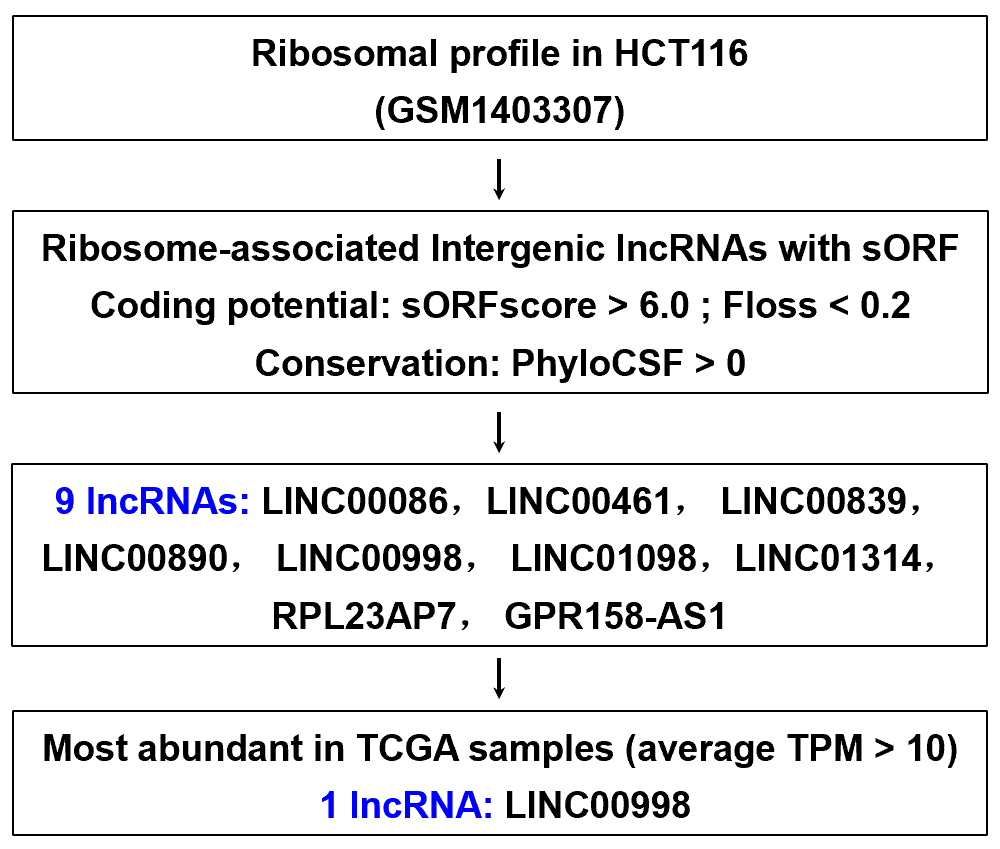


**Figure S1. The screening workflow for candidate lncRNAs with peptide-coding potential**. The ribosome-bound intergenic lncRNAs were retrieved from Gene Expression Omnibus (GEO) dataset (GSM1403307). The potential coding ability of the lncRNAs and the cross species conservation of the embedded sORF were evaluated using the online program ORFscore (http://www.sorfs.org)[1], which calculated a sORF score, a Floss score, and a PhyloCSF score for each lncRNA.

**Figure S2**

**Figure S2. Characterization of SMIM30 gene.** (A) Pan-cancer analysis of LINC00998 expression based on The Cancer Genome Atlas (TCGA) data. The relative expression levels of LINC00998 in paired tumor (T) and non-tumor (N) tissues from 11 different cancer types are presented. COAD, colon adenocarcinoma (n = 41); HNSC, head and neck squamous cell carcinoma (n = 43); KICH, kidney chromophobe (n =23); KIRC, kidney renal clear cell carcinoma (n = 72); LUAD, lung adenocarcinoma (n = 57); LUSC, lung squamous cell carcinoma (n = 49); PRAD, prostate adenocarcinoma (n = 52); READ, rectum adenocarcinoma (n = 9); STAD, stomach adenocarcinoma (n = 27); THCA, thyroid carcinoma (n = 58); UCEC, uterine corpus endometrial carcinoma (n = 23). Data are means ± SEM. ns, not significant by t‐test. (B) The PCR products of RACE assays. The products (indicated by red arrows) of 5’RACE (*left* panel) and 3’RACE (*right* panel) were obtained by PCR and then applied to direct DNA sequencing (n = 1). The nucleotide of the 5’- or 3’-end of SMIM30 transcript is marked by black arrows. (C) The sequence of the full-length LINC00998 RNA. The transcript consists of 3 exons, which are indicated by different colors. The small ORF is indicated with gray background, and the deduced amino acids are shown in blue letters. (D) Gene structure of LINC00998. The exons are indicated as white boxes, while the gray box represents the predicted ORF region. (E) The transmembrane domains in human SMIM30 peptide predicated by TMHMM program. Red thick lines represent the putative transmembrane domains, blue and pink thin lines indicate non-transmembrane regions. Inside, located within the cytosol; Outside, located outside the cytosol; TM-helicx, transmenbrane helix. (F) Schematic diagram showing the topology of SMIM30. It was predicted that SMIM30 contained two transmembrane helical regions, and both N- and C-terminus of SMIM30 are located in the cytoplasm.

**Figure S3**

**
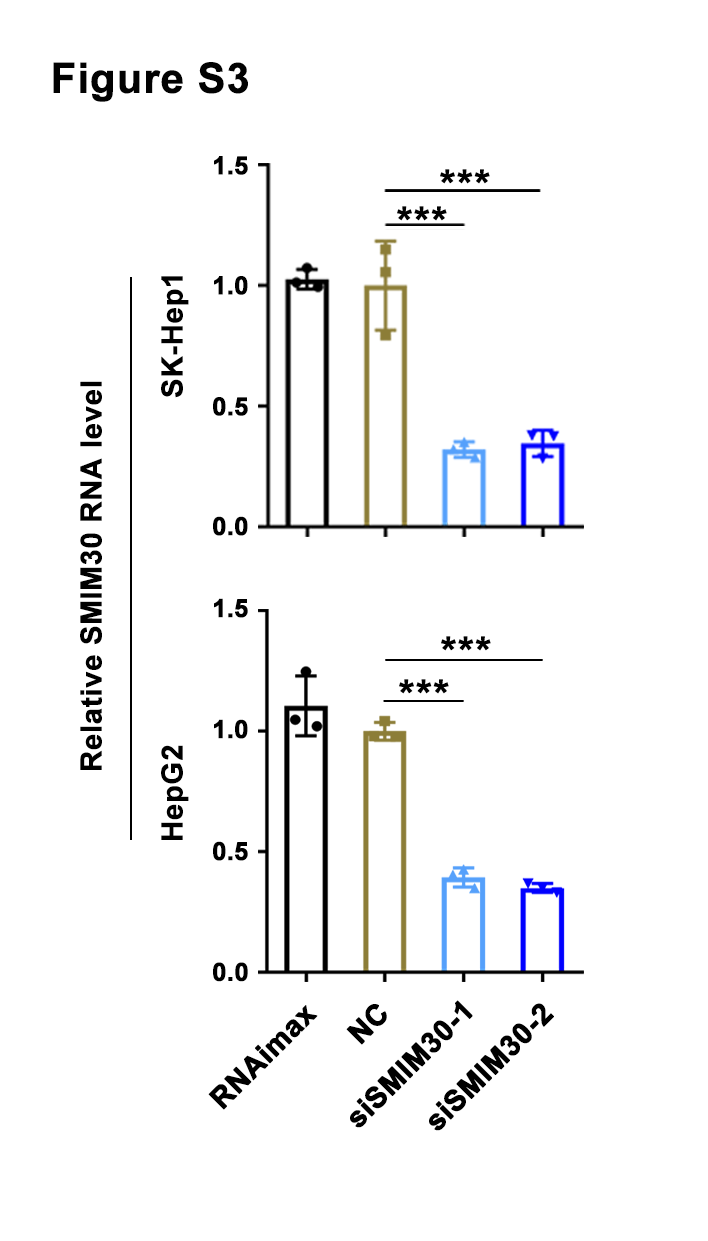
**

**Figure S3. Knockdown of cellular SMIM30 by siRNAs.** SK-HEP-1 and HepG2 cells were transfected with NC or siSMIM30 for 48 hours, then subjected to qPCR analysis. RNAimax, cells exposed to Lipofectamine RNAiMAX without RNA duplex; NC, negative control of RNA duplex; siSMIM30-1 and siSMIM30-2, siRNAs targeting different regions of SMIM30 mRNA. Data are presented as means ± SEM from three independent repeats, *** *P* < 0.001 by one-way ANOVA.

**Figure S4**

**
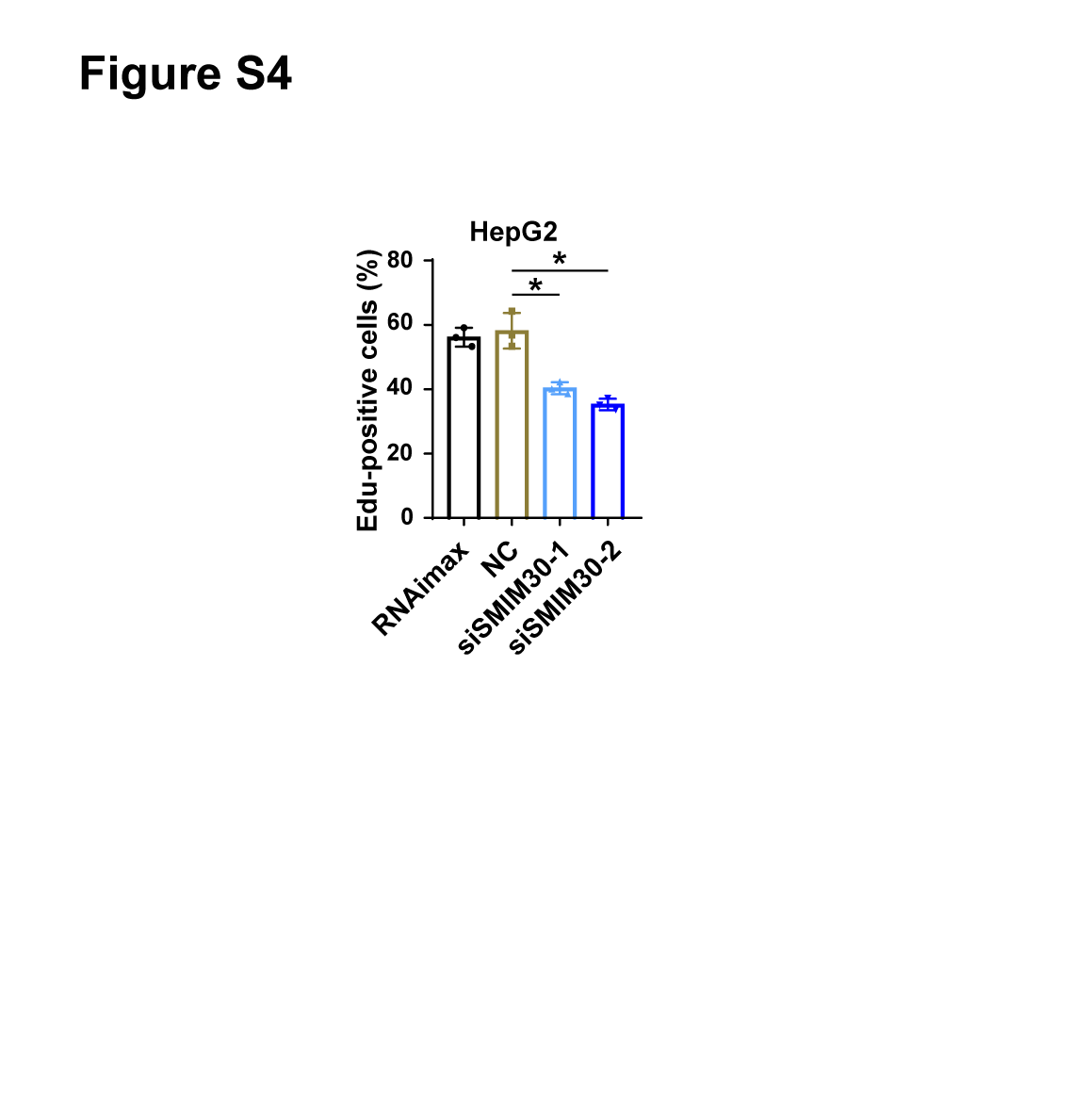
**

**Figure S4. Silencing SMIM30 reduced the fraction of cells with DNA replication.** NC- or siSMIM30 transfected HepG2 cells were serum-starved for 48 h, then cultured in 10% FBS-containing medium for 15 h before EdU incorporation assay. RNAimax, cells exposed to Lipofectamine RNAiMAX without RNA duplex; NC, negative control of RNA duplex; siSMIM30-1 and siSMIM30-2, siRNAs targeting different regions of SMIM30 mRNA. Data are presented as means ± SEM from three independent repeats, ** *P* < 0.01 by one-way ANOVA .

**Supplementary Tables**

Table S1. The levels of lncRNAs in different human tissues

| **genes**  **Tissue** | **LINC00998** | **LINC01314** | **LINC00839** | **LINC00890** | **LINC00086** | **LINC00461** | **LINC01098** | **RPL23AP7** | **GPR158-AS1** |
| --- | --- | --- | --- | --- | --- | --- | --- | --- | --- |
| **Bladder** | 38.48 | 0.3 | 0.53 | 0.09 | N/A^1^ | 0.04 | 0 | 7.13 | 0 |
| **Blood** | 143.12 | 0.05 | 0.5 | 0.02 | N/A | 0 | 0 | 9.18 | 0 |
| **Brain** | 17.26 | 3.57 | 4.08 | 0.01 | N/A | 15.55 | 0 | 6.22 | 0.72 |
| **Breast** | 27.54 | 0.18 | 0.93 | 0.05 | N/A | 0.01 | 0 | 7.39 | 0 |
| **Cholangial** | 16.1 | 3.95 | 0.05 | 1.35 | N/A | 0 | 0 | 1.13 | 0 |
| **Colon** | 22.99 | 0.46 | 0.48 | 0.45 | N/A | 0.35 | 0 | 6.56 | 0 |
| **Esophagus** | 26.13 | 0.08 | 0.66 | 0 | N/A | 0.02 | 0 | 4.29 | 0 |
| **Kidney** | 58.98 | 1.45 | 6.36 | 0.05 | N/A | 0.22 | 0 | 6.32 | 0 |
| **Liver** | 11.28 | 2.63 | 0.04 | 0.64 | N/A | 0 | 0 | 1.15 | 0 |
| **Lung** | 29 | 0.5 | 2.04 | 0.03 | N/A | 0.01 | 0 | 6.61 | 0 |
| **Ovary** | 17.71 | 0.29 | 0.96 | 0.21 | N/A | 0.19 | 0 | 5.91 | 0 |
| **Pancreas** | 9.96 | 0.16 | 0.07 | 0.01 | N/A | 0.01 | 0 | 1.47 | 0 |
| **Prostate** | 25.84 | 0.27 | 0.36 | 6.34 | N/A | 0.13 | 0 | 5.9 | 0 |
| **Stomach** | 16.07 | 1.04 | 0.1 | 0.04 | N/A | 0.04 | 0 | 2.64 | 0 |
| **Testis** | 15.57 | 0.52 | 0.46 | 0.02 | N/A | 0.37 | 1.76 | 7.78 | 0.05 |
| **Thymus** | 1.14 | 0.01 | 0 | 0 | N/A | 0 | 0 | 1.19 | 0 |
| **Thyroid** | 34.51 | 0.28 | 0.43 | 0 | N/A | 0.03 | 0 | 6.9 | 0 |
| **Uterus** | 23.91 | 0.16 | 1.23 | 8.41 | N/A | 0.34 | 0 | 8.6 | 0 |

^1^ N/A, not available.

The average gene expression level in each tissue is shown as mean transcripts per million (TPM) using the data of 18 normal tissues from TCGA database.

Table S2. Sequences of DNA and RNA oligonucleotides

| **Name** | **Sense Strand/Primer (5' - 3')** | | | | **Antisense Strand/Primer (5' - 3')** |
| --- | --- | --- | --- | --- | --- |
| **siRNA duplexes** |  | | | |  |
| siSMIM30-1 | UGAAAUGGCAACAAACAUAdTdT | | | | UAUGUUUGUUGCCAUUUCAdTdT |
| siSMIM30-2 | GCCCAGAAUCUUAGGAAAUdTdT | | | | AUUUCCUAAGAUUCUGGGCdAdA |
| NC | CUUACGCUGAGUACUUCGAdTdT | | | | UCGAAGUACUCAGCGUAAGdTdT |
| **Primers for qPCR** | | | | | |
| hsa-SMIM30 | CCTTAGTCCTCATGTCACTGC | | | | GCCTGTAATGCTGAGAACCA |
| Mmu-SMIM30 | AAGTTCAGCCCTCCAGATCC | | | | CTGGTCAGGGTGCAGTCTAA |
| CDC6 | AAGCTGTCTCGGGCATTGAA | | | | TGCCTTGCTTTGGTGGAGAA |
| DHFR | CACAAGGAGCTCATTTTCTTTCC | | | | AGTTTAAGATGGCCTGGGTGA |
| CCNE2 | CAGATAATCCAGGCCAAGAA | | | | CAGGCAAAGGTGAAGGATTA |
| GAPDH | GAGTCAACGGATTTGGTCGT | | | | GACAAGCTTCCCGTTCTCAG |
| β-actin | ACTGGAACGGTGAAGGTGAC | | | | AGAGAAGTGGGGTGGCTTTT |
| **Primers for RACE** | |  | | | |
| 3’RACE-adaptor-RT | ATGGCAGCAAGGTGATCACTAAAGTGATATCCTTTTTTTTTTTTTTTT | | |  | |
| 3’RACE-adaptor | | ATGGCAGCAAGGTGATCACTAAA | | | |
| 3’RACE-GSP1 | AGTCAAAGAAGTAAGATGGCTGA | | | |  |
| 3’RACE-GSP2 | GCCTCCTGAATCGTTGAGGAGTC | | | |  |
| **Table S2. Sequences of DNA and RNA oligonucleotides (Continued)** | | | | | |
| **Name** | **Sense Strand/ Primer (5' - 3')** | | | | **Antisense Strand/ Primer (5' - 3')** |
| 5’RACE-GSP | GATTACGCCAAGCTTGGAACCTTTCACACACCAAATCTCAGG | |  | | |
| **Primers for cloning into pcDNA3.0 (restriction enzyme sites are underlined)** | | | | | |
| pc3.0-ORF-GFP(△ATG) | CGGAAGCTTCTCGCCATTCCTGTAATGGCTGCT | | | | CTCGCCCTTGCTCACCATCTGTCCATTTCTTTTTCGTG |
|  | AGAAATGGACAGATGGTGAGCAAGGGCGAGGAGCTGTT | | | | TATGGATCCTCACTTGTACAGCTCGTCCATGCCGAG |
| pc3.0-SMIM30-GFP | CGG AAGCTTATGACCTCAGTTTCAACACAG | | | | CTCGCCCTTGCTCACCATCTGTCCATTTCTTTTTCGTG |
|  | AAAGAAATGGACAGAGTGAGCAAGGGCGAGGAG CTGTT | | | | TATGGATCCTCACTTGTACAGCTCGTCCAT |
| pc3.0-SMIM30-Flag | CTCGGATCCATGACCTCAGTTTCAACAC | | | | GCAGAATTCTCACTTGTCATCGTCATCCTTGTAATCCAT CTGTCCATTTCTTTTTCG |
| **Primer for cloning into pCDH (restriction enzyme sites are underlined)** | | | | | |
| pCDH-SMIM30-WT-Flag | TGCTCTAGAATTCCTGTAATGGCTGCTTCCT | | | | CGCGGATCCCTACTTGTCATCGTCATCCTTGTAATCCATCTGTCCATTTCTTTTTCGTGC |
| pCDH-SMIM30-FS-Flag | TGCTCTAGAATTCCTGTAATGGCTGCTTCCT | | | | GTGTTGAAACTGAGGCATGATGTTGGAATCTTGAGG |
|  | GATTCCAACATCATGCCTCAGTTTCAACACAGTTGTC | | | | CGCGGATCCCTACTTGTCATCGTCATCCTTGTAATCCATCTGTCCATTTCTTTTTCGTGC |
| pCDH-SMIM30-Flag | TGCTCTAGAATGACCTCAGTTTCAACACAG | | | | CGCGGATCCCTACTTGTCATCGTCATCCTTGTAATCCATCTGTCCATTTCTTTTTCGTGC |

**Supplementary reference:**

[1] Olexiouk V, Crappe J, Verbruggen S, Verhegen K, Martens L, Menschaert G. sORFs.org: a repository of small ORFs identified by ribosome profiling. *Nucleic Acids Res* 2016; 44, 324-329.
